# Ethical Concerns about Fashionable Dog Breeding

**DOI:** 10.3390/ani14050756

**Published:** 2024-02-28

**Authors:** David J. Menor-Campos

**Affiliations:** Departamento de Medicina y Cirugía Animal, Universidad de Córdoba, 14005 Córdoba, Spain; david.menor@uco.es

**Keywords:** fashionable dog, pure-breed dog, ethical breeding, dog breeding, dog abandonment

## Abstract

**Simple Summary:**

Humans have selectively bred dogs for various purposes, including hunting, guarding, and service roles. However, over time, preferences have shifted from functionality to aesthetics, resulting in a diverse range of dog breeds with varying sizes, shapes, and coats. Unfortunately, this focus on design and appearance has led to fad breeding, causing genetic disorders, health issues, and a loss of biodiversity. The article looks at fashionable dog breeding and questions the ethics of prioritising looks over health and behaviour. It aims to alert potential owners, breeders and regulators to the importance of considering a dog’s overall well-being, not just its appearance. Breeding brachycephalic breeds with respiratory conditions, inbreeding causing inherited disorders, and overbreeding popular breeds while shelter dogs remain unadopted raise ethical concerns. Furthermore, the impact of cosmetic surgeries on popular dog breeds, as well as the neglect of behavioural traits in favour of physical characteristics and strict breeding practices, are also considered problematic. The current breeding model can negatively impact the emotional and cognitive well-being of dogs. This can result in issues such as aggression, anxiety, and other behavioural problems which can significantly reduce the overall quality of life of the animals. Unregulated breeding practices and the demand for rare breeds can lead to illegal breeding, which compromises animal welfare. Prospective owners, veterinarians, kennel clubs, and legislators all need to play a responsible role in protecting animals.

**Abstract:**

The historical relationship between humans and dogs has involved selective breeding for various purposes, such as hunting, guarding, and service roles. However, over time, there has been a shift in preferences from functionality to aesthetics, which has influenced the diverse sizes, shapes, and coats of dog breeds. This review looks at fashionable dog breeding and questions the ethics of prioritising looks over health and behaviour. It aims to alert potential owners, breeders, and regulators to the importance of considering a dog’s overall well-being, not just its appearance, which has resulted in fad breeding, leading to genetic disorders, health issues, and a loss of biodiversity. Ethical concerns arise from breeding brachycephalic breeds with respiratory conditions, inbreeding causing inherited disorders, and overbreeding popular breeds while shelter dogs remain unadopted. Additionally, the impact of cosmetic surgeries on popular dog breeds, as well as the neglect of behavioural traits in favour of physical characteristics and strict breeding practices are also considered. The current breeding model can have a negative impact on the emotional and cognitive well-being of dogs, resulting in issues such as aggression, anxiety, and other behavioural problems that can significantly reduce their overall quality of life. Unregulated breeding practices and the demand for rare breeds can lead to illegal breeding, compromising animal welfare. Prospective owners, veterinarians, kennel clubs, and legislators all need to play a responsible role in protecting animals.

## 1. Introduction

The relationship between human and dogs has a long history, of more than 100,000 years [1], whether humans deliberately removed young wolf pups from the den and hand-reared them as dependents, or a more unlikely situation, in which certain wolves might have decided to keep close to those primates which leave a good amount of food behind (see [2] for a recent discussion on the topic). This relation has shaped our species, helping it to evolve, in the same way that our ancestors, and latter ourselves, shaped other species through domestication [3].

In the early times, dogs probably assisted humans with hunting, guarding, and scavenging, playing a vital role in human survival by alerting us to danger, tracking game, and even participating in cooperative hunting [4]. As humans transitioned from nomadic hunter-gatherer societies to settled agricultural communities, they also helped guard livestock, or protect crops. Later, they would have acted as guardians of human settlement, bringing alarm to intruders or strangers. Linked to power and royalty, they would end up appearing in the mythology, art, and religious practices of many ancient civilizations [4,5]. Our relationship is one of the oldest and most significant human–animal bonds in history, thanks to the remarkable adaptability of dogs and their deep integration into human societies [6].

Over time, human ancestors first, and human beings later, selected dogs for specific traits, from the more sociable or at least manageable, to faster in the run and brave in the hunt, seeking to fulfil different and changing tasks as the time went by, leading to the emergence of breeds [7].

Being the very first species that our human ancestors domesticated has tied dogs to human society seemingly for ever. Nevertheless, domestication, alongside all these processes and all these selection procedures comes at a cost. This new species, dogs (*Canis lupus familiaris*), lost several traits, abilities, and shapes, among other characteristics, in their way to our homes. Dogs are less trouble solving than their wolverines ancestors [8], and often retain juvenile traits and behaviours into adulthood, a phenomenon known as neoteny [9], which might make them more human dependent, and thus desirable. On the other hand, dogs have become more tolerant, decreasing their aggressiveness and increasing their playfulness, which facilitates social interactions and enhances the human–dog bond, while adapting to live closely with humans in a wide variety of environments and situations [10,11].

However, as the value of dogs as companion animals increases and their ownership becomes more commonplace in western culture, the popularity of certain traits in fashion breeds has raised concerns about the ethical implications of prioritising appearance over health. Instead of researching and selecting a breed that suits a particular lifestyle, or even considering adoption from shelters and rescue organizations, some owners choose their pet based solely on its physical appearance, treating it as a fashion accessory, and even ignoring the potential welfare problems related to extreme conformation and inherited disease [12,13]. Although many disorders and gene variants in dogs are shared among closely related breeds due to the historical population dynamics of this species, many disorders are specific to certain breeds [14], and they are often linked to distinct morphological characteristics of these breeds [15].

In this scenario, commercial dog breeding, especially of fashionable breeds (those that become commercially most popular in a very short time), can lead to a breakdown in the human–dog bond, as dogs are treated as commodities rather than valued companions [16].This, in turn, would mean insufficient consideration of dogs’ welfare needs and interests, leading to potential harm and suffering [16]. An increasing number of dogs are purchased worldwide each year. However, a lack of data on the conditions faced by dogs in commercial breeding kennels, during transport, and after they are discarded impedes a comprehensive understanding of the extent and nature of the issues, as well as potential solutions. Breeding dogs solely for their physical appearance in order to increase profits is itself a real form of animal abuse [17]. Additionally, the conditions in which dogs are bred are often harmful, with overcrowding and unsanitary environments leading to lifelong mental and physical health issues. Plus, a selective breeding focus on physical traits only can lead to a decrease in the genetic variability of the species, an accumulation of genes associated with pathologies, and an exaggeration of physical traits to levels that may compromise health [18,19]. Selecting dogs based solely on their physical appearance implies that an unknown number of discarded dogs that did not meet expectations, even though they may not appear in statistics and nobody knows what their fate is.

Therefore, this article presents current information on fashionable dog breeding and explores and analyses the ethical issues connected with prioritising physical appearance over health and behavioural well-being in order to spark the concerns of prospective owners, breeders, and regulators by highlighting what is behind the dog they choose.

## 2. Historical Context

### 2.1. The Very First Dog Breeds

Even though this seems to be a debate that is still open, there are several breeds that could be considered to be among the oldest dog breeds in the world. The Greyhound breed is considered to be one of the oldest purebred domesticated dog breeds [20], with indications of its existence dating back to the era of the Pharaohs, and the earliest documented records of greyhound-like dogs originating from about 8000 years ago [21]. This breed is closely followed by Basenji [20], a breed that is believed to have originated in Africa, dating back to around 6000 BC, with Libyan cave paintings that depict the hunting dog as a time reference. Following this, the Saluki breed, which has a long and ornate history with remains tracing to ancient civilizations such as the Egyptians, Sumerians, and Persians, with archaeological evidence dating back at least 5000 years, comprised a sighthound developed from the Fertile Crescent and used by nomadic tribes to hunt by sight [22]. Other breeds around this history time are the Akita Inu, believed to have been originated in Japan more than 5000 years ago [23]; the Siberian Husky and Alaskan Malamute, deriving 4000 years ago [23]; the Afghan Hound, 3000 years ago [23]; and the Chow Chow and Shar Pei, 2000 years ago [22].

In addition to the exact point at which these breeds appeared, what seems to be common in all of them is that they all would have been selected because of their endurance capacity or their abilities for hunting in different part of the world [22,23].

### 2.2. The Modern Breeds

The physical variability in domestic dogs is remarkable, as is their size, with striking differences between the miniature and the giant breeds [24]. This extensive phenotypic diversity arose through thousands of years of artificial selection and reached its peak in the creation of hundreds of breeds over the last two centuries [25].

Still, this selection is still on the run. As societal and human needs have changed, the role that dogs play has changed as well, passing from purely hunting dogs to being used for transportation, guarding, herding, police dogs, search and rescue dogs, more recently as service animals for individuals with special needs, but also as a luxury value or a fashion good. In these cases, aesthetics heavily influence dog choice and, therefore, breed conformation, leading to an industry primarily focused on a dog’s appearance [26], as owners shift from prioritising the animal’s utility to emphasising its visual appeal [13]. There are over two hundred breeds—the Federation Cynologique Internationale recognises 356 breeds [27], meanwhile the American Kennel Club 200 breeds [21], each of them with an established breed standard which specifies precise phenotypical ground regarding colour, height, or body shape alongside other specific physical features such as tail length or ear positioning, which might even require surgical interventions. Appearance is not everything. But it does matter.

### 2.3. Neoteny, Problem-Solving Skills, Independence, and Maturity

Certainly, thousands of years of selection and domestication have influenced dogs’ behaviour, making them more inclined to be in close proximity with humans [28]. This has also facilitated their ability to cooperate and communicate with humans [29,30,31], interpreting owners’ gestures, such as gaze, cues, or pointing [31,32], or even being able to understand humans’ attentional states [33,34]. But they have also lost something in the run.

Neoteny [9] is the term that describe the retention of juvenile traits into adulthood, based on human preferences [35]. It is a common evolutionary phenomenon linked to domestication that significantly influences the physical appearance—childish—and behaviour—friendly—of animals throughout their domestication journey, and has been a driving force behind the remarkable diversity in the dog breeds that we witness today. Thus, selective breeding for certain traits, such as a more youthful appearance, smaller size, and altered physical features, has made dogs more endearing and visually appealing to humans over thousands of years, playing a role in the likelihood of dogs being allowed to breed, adopted, and cherished as companions [35,36].

However, the prolonged retention of juvenile characteristics in dog breeds has led to concerning health ramifications, such as the size and shape of their heads [37], extreme alterations in body structure [38,39], or even alterations in their behaviour [40].

In addition, neoteny also affects the pace of sexual maturation in dogs. Domestication is known to cause a general acceleration of sexual maturity in animals [9], and this phenomenon occurs also in dogs [41,42,43], affecting their behaviour [44] and health [18].

On the whole, many dogs seem to have lost their problem-solving skills, independence, and maturity in exchange for a stable source of food and care compared to wolves. Although some dogs still have problem-solving skills, such as earthquake dogs, as well as drugs and weapons dogs in airports, the average dog seems to struggle when confronted with a challenge. When faced with challenging tasks or puzzles, wolves tend to persevere and actively engage with the problem until they find a solution, while regular dogs often display a tendency to seek human intervention or assistance when encountering difficulties [31]. Though this reliance on human guidance might stem from their long history of domestication and dependency on humans for survival, it might have impacted their independent problem-solving capabilities [45].

Moreover, studies focusing on the social dynamics and cooperation within wolf packs versus dog groups have highlighted intriguing differences, as wolves have been found to display higher levels of prosocial behaviour compared to dogs in certain contexts. This distinction in social behaviour further reflects upon their problem-solving strategies. Wolves, being more prosocial, might rely on each other within their pack, sharing information and collaborating to solve problems, whereas dogs might be more inclined to seek assistance from humans due to their dependency [46].

## 3. Genetic Hazard

### 3.1. Increased Risk of Inherited Disorders

Breeding for specific traits may unintentionally increase the likelihood of genetic disorders [18], making them sadly common among popular dog breeds [13]. For instance, Border Collies, which are among the world’s most popular breeds, are known to have at least 25 inherited disorders [47]. Table 1 shows a non-comprehensive list of inherited disorders in popular dog breeds.

This breeding practice also results in diminished genetic diversity, the accrual of detrimental genes, and the amplification of specific physical traits, consequently elevating the health risks they face [98,99]. Mellanby [100] explains this phenomenon through the concept of a ‘breed barrier’ that encourages inbreeding, as animals need to have five previous generations of ancestors registered as the same breed before they can be registered as a pure-breed dog, promoting reproductive isolation.

Additionally, as breeders prioritize the appearance of their dogs to meet specific physical breed standards, as approved by organizations such as kennel clubs, they can obscure the genetic predisposition of these dogs to various illnesses and physical issues. In 2009, Asher et al. [26] conducted a study documenting 396 inherited disorders within the top 50 breeds in the United Kingdom. These disorders affect a variety of systems, including musculoskeletal, integumentary, nervous sensory, cardiovascular, urogenital, respiratory, gastrointestinal, immune, and endocrine; and their frequency showed a correlation with the registration numbers of specific breeds, indicating an increased likelihood of such disorders with continued breeding of the same dog breeds.

Distinctive traits can often differentiate one breed from another, but these same traits can also be direct triggers for medical issues that persist throughout a dog’s life. For example, certain breeds with smaller cranial cavities may be more susceptible to severe neurological conditions such as syringomyelia [90]. One of the most frequently observed conditions among these is Chiari-like malformation/syringomyelia in the Cavalier King Charles Spaniel. This breed is also susceptible to various neurological conditions, such as fly catching [90], idiopathic epilepsy [91], idiopathic facial nerve paralysis with or without associated vestibular disease [92], and degenerative myelopathy [82], all of which are linked to the shape of their lovely skull.

Breeds with curly tails, such as Pugs and Basenjis, are prone to spinal issues like hemivertebrae and spina bifida due to the nature of their tails [26,93]. The Rhodesian Ridgeback is named after the distinctive ridge of hair along its lower back, which stands in the opposite direction with two whorls near the head. Regrettably, the name of this breed is linked to dermoid sinus, a developmental abnormality that creates tubular indentations in the skin above the spine. These tunnels can penetrate deep into underlying tissues, even reaching the spinal cord, exposing affected animals to infection risks that cause severe disease and pain. Salmon Hillbertz and Andersson [94] have confirmed the inheritance of the dorsal ridge in Rhodesian Ridgebacks through an autosomal dominant mode, linking it to dermoid sinuses. Although not all Rhodesian Ridgebacks are born with this ridge, breed standards mandate its presence [101]. Furthermore, this breed is known to suffer from arrhythmia or sudden death, which may be associated with an autosomal recessive pattern of inheritance [95].

Deafness in Dalmatians is linked to the extreme piebald gene [96], which is responsible for the majority of their coat’s whiteness and, in some cases, their blue eyes. Dalmatians exhibit a homozygous recessive trait for the extreme piebald gene, and deafness appears to manifest as a linked polygenic disorder. Inheriting this gene significantly increases the likelihood of also inheriting genes that cause deafness [97]. However, some Dalmatians may exhibit incomplete penetration of the extreme piebald gene, resulting in larger patches of dark fur than the typical spots as well as a lower risk of deafness [102]. Unfortunately, these patches are considered flaws or faults according to the breed standard.

More broadly speaking, Asher et al. [26] have linked dog size and several conditions, stating that taller dog breeds are more prone to cardiovascular, gastrointestinal, integumentary, and musculoskeletal disorders, such as hip and elbow dysplasia, which are often linked to their larger body size and rapid growth. Conversely, lighter breeds exhibit a greater incidence of respiratory, urogenital, and endocrine disorders. In contrast, smaller breeds are at a higher risk of developing nervous sensory, respiratory, and urogenital issues, as well as specific conditions like odontoid process dysplasia, shoulder dysplasia, and patellar luxation. Additionally, smaller dogs are particularly susceptible to conditions such as patellar luxation, which can lead to lameness. In addition, these authors also highlight that breeds with brachycephalic, short skulls may experience respiratory challenges due to their skull structure, including dyspnea, stenotic nares, elongated soft palate, and hypoplastic trachea. Selecting breeds to produce dogs of such extreme sizes, whether giant or dwarf, would push forward all these related conditions.

The Universities Federation for Animal Welfare [103] operates a website (https://www.ufaw.org.uk/dogs/dogs (accessed on 10 January 2024)) that provides information to prospective owners about genetic welfare problems in companion animals. The website details health issues related to genetic conformation in over 40 dog breeds.

### 3.2. Challenges of Physical Traits

Evidence suggests that physical appearance outperforms health considerations when acquiring a dog [104,105]. Too often, body size [106] or hair type and length [107] were prioritised over health and longevity [108]. Indeed, the most popular breeds usually have significant health issues [13]. Breeds such as Bulldogs, Pugs, and French Bulldogs are often admired for their cute and ‘pushed-in’ appearance, characterized by short snouts and flat faces, following the Lorenz’s ‘baby schema’: Infantile facial features elicit positive emotions and nurturing responses in human adults [109]. Cultural factors and anthropomorphism might have affected the demand for these characteristics, referred to as brachycephalic features. Animals with flat faces, wide-set eyes, truncated snouts, and rounded faces tend to elicit feelings of endearment and cuteness, reminiscent of a baby-like charm. This is a distinctive appearance that can foster a sense of protectiveness and care among humans [110].

Media forms such as advertisements, movies, cartoons, and social platforms can significantly influence public perceptions and preferences [13,111,112]. However, this conformation can cause brachycephalic obstructive airway syndrome (BOAS, see [37] for a recent review of this syndrome), with a high predisposition to a range of disorders intrinsically related to this conformation, including respiratory disease [39,65,113], eye disease [39,113,114], dystocia [65], spinal disease [115], or even exercise intolerance and heat stroke [113], alongside a higher prevalence of health problems thought to be unrelated to conformation, such as skin cancer [113], and a shorter lifespan compared to moderate and non-brachycephalic dogs [114]. The situation is so dramatic that professionals have warned against the unsustainable breeding of Bulldogs, Pugs, and French Bulldogs due to their health and welfare issues, yet these breeds remain popular with owners [116].

Breeds like Pekingese, Shih Tzus, and Cavalier King Charles Spaniels are known for their large, expressive eyes, which can convey a wide range of emotions, including happiness, curiosity, innocence, and vulnerability, and they have been used even in various forms of art and illustration to convey emotion, personality, and character depth [117]. As humans often connect or empathize with animals that display emotions similar to their own, having this trait will reinforce our willingness to interact with these animals [118,119,120]. Furthermore, as humans are a visual species, expressive eyes can create a sense of understanding and bonding, as people interpret the emotions behind the gaze [104].

The problem is that these dogs with large, bulging eyes are more susceptible to eye injuries, infections, and conditions such as corneal ulcers. Breeds with flat faces, such as Pugs and Boston Terriers, are also at a higher risk of eye issues and injuries, including cherry eye (prolapsed gland of the third eyelid), due to the shape of their eyes [121]. These dogs frequently have wide eye openings and shallow eye sockets, which causes their eyes to protrude more than usual [122]. This unique eye structure can make them susceptible to damage from external sources and even prevent them from fully closing their eyelids [122,123], resulting in inadequate blinking (lagophthalmos). This inability to blink properly compromises the protective tear film, leading to dry areas on the cornea that can eventually erode and cause ulcers [124].

But it is not only flat-faced or wide-eyed dogs that are people’s preferences. People are drawn to Dachshund dogs for their unique appearance, playful personality, adaptability, and historical significance [125]. Dachshunds, often referred to as “wiener dogs” or “sausage dogs”, have a distinctive elongated body and short legs that make them stand out in a crowd and contribute to their charm and individuality, creating a comical and adorable appearance that appeals to many people’s sense of humour. Initially selected for hunting, Dachshunds have been featured in various forms of media, including movies, TV shows, advertisements, and social media, which might have influenced people’s choices [13,111]. In addition, they are also known for their lively and playful personalities. Despite their determination and independence, they are often curious and energetic while enjoying being close to their family members, forming strong bonds with their owners, and developing a loyal and affectionate connection. Therefore, they have captured the hearts of many dog lovers, despite their specific health considerations due to their elongated body structure, such as intervertebral disc disease (IVDD) [126] and spinal issues. Though IVDD can affect dogs of various breeds, it is more commonly seen in those breeds with longer backs and shorter legs, such as Dachshunds, but also Beagles, Corgis, and Basset Hounds [38,39]. The spinal discs act as cushions between the vertebrae and allow for flexibility and shock absorption in the spine. IVDD occurs when the inner, gel-like material of a spinal disc herniates or ruptures through the tougher outer layer, causing compression on the spinal cord or nerve roots [126]. The consequences of IVDD and spinal issues in dogs can vary depending on the severity of the condition and the location of the affected disc, but they can cause varying degrees of pain and discomfort, from stiffness to reluctance to move and a hunched posture, compression of the spinal cord or nerve roots, and can lead to neurological symptoms such as weakness, wobbliness, loss of coordination, causing difficulty to walk or even stand [127,128].

While some cases of IVDD can be managed without surgery through conservative measures such as rest, pain medication, anti-inflammatory drugs, and physical therapy, severe cases of IVDD can lead to complete paralysis of the affected limbs or even the entire hindquarters, and dogs may lose control over their bladder and bowel functions, impacting dogs’ quality of life and requiring surgical intervention to remove the herniated material and stabilize the affected area by alleviating pressure on the spinal cord and nerve roots [128].

Other joint and skeletal issues, such as hip or elbow dysplasia, are also common in breeds like Bulldogs, Basset Hounds, and Dachshunds due to their unique body shapes, leading to chronic pain, mobility issues, behavioural problems related to aggressiveness, and compromised welfare [129].

Other aesthetic choices, such as the wrinkled skin in dogs like Shar Peis and Bulldogs, can contribute to a breed’s charm, but these features can also lead to skin infections and irritation within the folds [130]. Wrinkled skin folds can trap moisture, dirt, and debris, making regular cleaning and maintenance necessary to prevent skin infections [131]. This may set certain breeds apart from others, making them easily recognizable and distinct and attracting people by their uniqueness. People may find the texture of the skin interesting and enjoyable to touch, as it provides a different tactile experience when petting or interacting with the dog. The soft and pliable nature of wrinkled skin can evoke feelings of comfort and tenderness. Furthermore, the folds and creases on a dog’s face can create comical and expressive facial expressions and can evoke emotions like amusement and warmth in humans. But none of these reasons can be more important than the health of the dogs themselves.

### 3.3. Loss of Biodiversity

As breeders of fashionable dogs prioritize specific physical traits, such as coat colour, size, shape, or other distinctive features, they cause the selection of a narrow range of the genetic diversity within the breed, contributing to the loss of biodiversity within dog species. To obtain the desired appearance traits, breeders usually rely on close inbreeding or breed from a limited number of individuals with the desired traits, which creates a kind of genetic bottleneck accompanied by a consequent reduction in the gene pool and, therefore, a loss of genetic diversity [132]. Furthermore, this can have negative consequences for the health and well-being of the dogs by increasing the likelihood of passing on undesirable traits, including genetic disorders, to the next generation while ignoring the breed’s original functional traits or working abilities.

Genetic bottlenecks and the drastic decrease in the genetic variation in populations are often associated with events such as natural catastrophes or epidemics. In the case of the dog, Machová et al. [133] have attributed their occurrence to breeding from a very small number of parents. In some breeds, such as the Border Collie, 50% of their genetic variability is linked to fewer than ten animals [134].

While the number of individuals in the different breeds has grown over the last thirty years, their inbreeding coefficient has doubled, and an average of 70% of the genetic variability has been lost [135]. Inbreeding could easily have been avoided, judging by the number of dogs registered as purebred, but genetic analysis confirms that this has not been the case. In fact, dogs have lower intraspecific variation than humans or mice [136].

Both inbreeding and genetic bottlenecks can lead to a population of dogs that look and behave similarly, as breed standards request, but they also have negative consequences for a breed’s health and well-being: inbreeding can concentrate recessive genetic disorders, leading to higher rates of inherited health problems [26,121] due to an increased load of deleterious variation [137]; reduced genetic diversity can compromise the breed’s ability to resist new diseases and adapt to changing environments [135].

Responsible breeding practices that prioritize genetic diversity, health, and overall well-being are crucial to mitigate the negative effects of inbreeding and genetic bottlenecks. Genetic testing, outcrossing (breeding with unrelated individuals), and careful selection of breeding partners can help maintain the long-term health and viability of dog breeds. Otherwise, the popular sire phenomenon will spread inherited defects [99].

### 3.4. Behavioural Divergence of Breeds

Selective breeding can have a significant impact on the behaviour of animals, as well as their overall health and well-being. Interestingly enough, popular breeds are not necessarily better in terms of behaviour, lifespan, or health outcomes. In fact, Ghirlanda et al. have found that popular dog breeds tend to be less trainable and more prone to separation anxiety, fear of other dogs, and aggression towards their owners [13].

Given that animal behaviour is a significant factor in abandonment or relinquishment to shelters [138], it should be expected to be one of the more interesting issues to be considered by breeders and breed standards. However, Asher et al. [26] suggest that aesthetics play a more significant role in the industry’s focus, with appearance taking precedence over behaviour. Indeed, breeds can become popular despite problematic behaviour rather than because of good behaviour [13].

Dogs’ behaviour and temperament are formed, conditioned, and modulated both by genetics and life experiences [139]. Therefore, all those awkward situations that dogs suffer in commercial breeding facilities will shape their behaviour.

Selective breeding has also influenced the genes associated with behaviour [140], and the degree of selection has also impacted behaviour [141]. The observed behavioural differences suggest that dog breeds have diverged not only morphologically and genetically but also behaviourally [142].

Previous studies have identified breed variations in noise sensitivity [143,144,145] and fearfulness [146,147,148,149,150,151,152].

While certain breeds which have been selected for hunting, such as Pointers, seem to tolerate sudden noise better [143,145], other breeds selected for herding or even cross-breed dogs showed a high sensitivity to noise [153]. Whether a random effect—popular sires, genetic drift—or a correlated one linked to other desiderated traits, the alleles of anxiety genes seem to have been accumulated in specific breeds during selection [145].

Wright et al. [154] have found that smaller breed dogs tend to show higher impulsivity levels than larger breed dogs, which they explain is due to owner efforts to control dogs of a considerable size to prevent worse problems; in comparison, owners show indulgence towards small dogs’ activities because the consequences or the perceived risk is minor.

Research has also found a correlation between a dog’s size and its likelihood of exhibiting aggressive behaviour. Smaller dogs have been shown to be more prone to aggression, as well as other undesirable behaviours such as fear [142,155,156,157,158,159,160,161]. Aggression and fear are associated with the same genetic loci linked to small body size, as suggested by the correlation found in Zapata et al.’s studies [162,163].

As the breeding of dogs can have an impact on their mental health, certain breeds may be more susceptible to issues such as fear of other dogs, separation anxiety [142], and sensitivity to touch. Duffy et al. [164] have found significant breed differences in aggression levels, where those breeds that are bred for guarding, protection, or fighting purposes may have a higher likelihood of displaying aggressive behaviours. But they also found that aggression is not solely dependent on breed but is influenced by individual variation within breeds as well, which highlights the importance of considering an individual dog’s temperament and behaviour when choosing the sires.

Additionally, studies have shown that certain behaviours, such as trainability, aggression towards strangers, attention seeking, and attachment, may be more heritable than others [165]. Col et al. [144] have investigated abnormal repetitive behaviour, and Dinwoodie et al. [147], Flint et al. [166], Hsu and Sun [167], McGreevy et al. [160], Serpell and Duffy [149], and Takeuchi et al. [168] have explored aggression, while Takeuchi et al. have studied separation-related behaviour [168].

Most studies have difficulty establishing a definitive correlation between dog breeds and certain behaviours due to sample characteristics. Some breeds are more prevalent than others, while some are underrepresented. Additionally, outcomes may be blurred by differences in background, life experience, and ownership. Furthermore, it was not investigated whether the specific behaviour was due to the breed’s genetic makeup or a particular line of ancestors.

## 4. Irresponsible Dog Breeding

### 4.1. Packed Shelter Dogs: Overbreeding and Abandoment

Dogs exhibit high fertility rates, encompassing pregnancy rates, birth rates, and litter sizes, while experiencing relatively low abortion and stillbirth rates, although variations exist among breeds [169]. The combination of elevated fertility and a robust demand for dogs makes breeding a lucrative venture. However, this has led to overproduction, contributing to the overcrowding of animal shelters and the euthanasia or killing of millions of dogs annually, either due to waning interest or economic considerations.

The emergence of third-party sales, notably through “puppy mills”, has begun in certain countries like the UE, the USA, and Australia. Nevertheless, the proliferation of online platforms and direct buyer interactions complicates regulatory control, which is exacerbated by the absence of stringent regulations governing the trade of pedigree dogs. Little is known about the fate of dogs discarded during the selection process for new traits, or the numbers of litters rejected for not being big, small, flat faced, fluffy haired, or cute enough, alongside retired breeding females and leftovers. However, these animals, deemed as low-value commodities, may end up roaming the streets or overcrowding shelters.

Estimates from the European Union suggest that around 100 million abandoned companion animals, with the majority residing in member states, contribute to the persistent issue of pet overpopulation [170]. In the U.S., the ASPCA estimates that approximately 3.1 million dogs enter animal shelters annually, with 390,000 dogs being euthanized each year [171]. The most common source of dog acquisition is from breeders, accounting for 34% in contrast to the 23% sourced from shelters [172].

While the duration of a dog’s stay in a shelter varies widely [173], the lack of euthanasia policies results in the development of long-term dog populations, particularly for older, male, large-sized, neutered dogs of a “dangerous breed” displaying behavioural problems such as aggression and high arousal [174,175].

Dogs placed in the shelter environment face various stressors, such as disruptive sounds, movement restrictions, and the loss of social connections [176]. Prolonged shelter stays can negatively impact dogs, leading to increased signs of aggression, restlessness, and difficulty relaxing, as observed in long-term-stay dogs [175]. Chronic stress-related behaviours, including increased aggression, excitement, uncertainty, paw lifting, vocalizations, repetitive behaviours, circling, self-licking, panting, and holding the head up during rest, may manifest over time [177,178,179,180]. The presence of these behaviours reduces the likelihood of dogs being adopted, creating a negative cycle where they spend more time in shelters and are less likely to be rescued while thousands of potential owners worldwide are looking for a new puppy.

### 4.2. Dogs as a Value Product: Inbreeding and Puppy Mills

The business model is founded on three key principles: ensuring supplies align with demand, maximizing production, and minimizing costs. Though these issues are already prevalent in many conventional commercial dog-breeding programmes, they are even more pronounced in the context of fashionable breeds.

The popularity of a breed, its fluctuations, and the rates of increase and decrease around popularity peaks are not necessarily aligned with inherent breed characteristics. Instead, these parameters are primarily influenced by trends and fashions rather than functional considerations [13]. For instance, showcasing a dog as a heroic character in a film can result in a substantial increase in breed registrations for that specific breed, and this trend may persist for up to ten years after the movie’s release [181].

Once the demand exists, the supply must be delivered quickly, so a breeder will try to bring certain dogs to market. But the problem is that when you are looking for a dog with specific traits, you have to control which dogs are the parent, and the parent of the parent, and the parent of them, and so on as long as possible, to avoid some recessive gene showing up in the litter of your interest while ensuring that the desirable traits will be present in them. At this point, breeders have two main options. On one hand, they could spend time and money checking the DNA of the potential parent, so as to ensure that neither stranger nor hazardous genes can pass to the precious descendent. On the other hand, they could turn to dangerous practices, such as inbreeding.

The term ‘inbreeding’ originated from the Victorian practice of intentionally introducing a novel trait, such as a curled tail or a specific coat pattern, by consistently breeding dogs that exhibit that particular characteristic. This process often commenced with crosses between parent and offspring or among siblings [182]. This breeding method enables the rapid fixation of significantly altered traits within a population. However, several generations of inbreeding aimed at stabilising a particular trait can lead to offspring that are predominantly homozygous. In addition to the actively selected traits, other alleles and characteristics tend to rise to high frequency during this process, a phenomenon often referred to as ‘hitchhiking’, which may occur due to the physical linkage between genomic regions that regulate multiple traits [183] or because of the pleiotropic effects of the selected trait. And in many cases, this also increases hereditary pathology and conformational disorders, such us orthopaedic and joint disorders [26,184,185]; skin disease [26]; aural disease [186]; ocular disease [121,187]; or even breathing difficulties, like the sadly famous brachycephalic obstructive airway syndrome (BOAS) [121,188,189].

In numerous dog breeds, the effective population size remains relatively small due to tightly controlled breeding within closed populations [99]. Dogs that are not intended for breeding purposes are often neutered, and human intervention in selecting and controlling breeding pairs further reduces the reproductive population compared to the census population. Population reductions often result in a significant loss of genetic diversity due to genetic drift, which can lead to increased homozygosity and a higher risk of inbreeding depression. This can reveal recessive harmful traits or occur through over-dominant genetic positions, where the hybrid form exhibits superior fitness. Inbreeding depression can also lead to reduced fertility, such as sperm abnormalities [190], and an increased incidence of congenital diseases [191,192].

Furthermore, inbreeding can have a negative impact on lifespan, as it has been observed across various species, from fruit flies [193] and butterflies [194], to cattle [195] or dogs [196,197].

As the phenotypic variation in dog species is widely recognized, there is significant diversity in the mean genomic inbreeding and the frequency of deleterious alleles among dog breeds, with larger breeds tending to exhibit higher levels of inbreeding compared to smaller breeds [197]. These variations in inbreeding do not appear to be related to the current size of the breed population. Instead, they are associated with differences in inbreeding strength at the time of breed creation, such as founder effects, and variations in modern or historical breeding practices, such as the use of popular sires.

Inbreeding practices are frequently linked to puppy mills, which are commercial farming operations that breed large numbers of purebred dogs [198]. These ‘puppy farms’ prioritise the production of a large number of animals over the welfare and quality of their puppies. They have no qualms about breeding indiscriminately, in conditions of constant confinement with little or no socialisation, human contact, or veterinary care [199].

Conditions in these facilities can vary from clean and well-maintained to unsanitary and harmful to animal health and welfare [200,201,202]. They often house a large number of dogs and aim to maximize space within legal limits. Dogs bred for reproduction are frequently confined to cages throughout their reproductive lives, with little opportunity for exercise or positive human interaction, and their healthcare needs are often neglected [203,204].

Dogs need sufficient space for movement, play, and exploration, which are essential for their physical and mental well-being [205]. They are sociable animals and require regular social interaction and exercise [206,207,208]. Animal welfare risks in such conditions arise from confined spaces, inappropriate group sizes, exposure to artificial light, loud or distressing sounds, strong odours, uncomfortable temperatures, and unsuitable flooring. These conditions can result in health hazards, hindrance in displaying natural behaviours, restricted movement, disrupted resting patterns, sensory overload, stress from isolation, and an inability to engage in playful behaviour [209,210].

Prolonged confinement and insufficient social stimulation contribute to the development of behavioural problems and a poor level of welfare in dogs [211]. They tend to exhibit behavioural and psychological irregularities compared to the general population of pet dogs [203,212], such as in stereotypical behaviours [213].

These dogs also tend to develop intense and lasting fears, potential learning difficulties leading to lower trainability, and often struggle to adapt to normal life, showing anxiety, house-soiling behaviour, and compulsive behaviours even after years [214].

The lack of an adequate socialisation and exposure to environmental stimuli, which are common in commercially bred dogs, may result in the development of what is commonly referred to as ‘kennel-dog syndrome’, characterized by the animal exhibiting fear and timidity in unfamiliar social situations or environments [215,216]. They might also display abnormal behaviours such us compulsive or repetitive behaviours, reduced trainability due to cognitive deficiencies, or an inability to form proper connections with humans [157,217,218], alongside a high sensitivity to touch and increased reactivity [5].

This experience is even worse for puppies, and they might be affected even from their gestation. The psychological development of the foetus can be significantly impacted by the circumstances and experiences during the prenatal phase of breeding dogs in ‘puppy mills’. Research has shown that offspring from pregnant animals exposed to stressors may exhibit neurohormonal dysfunction [219] and disruptions in the regulation of the HPA axis [220,221,222]. Offspring may exhibit abnormal responses to stress [223], increased sensitivity to stressors [224], and difficulties in coping with stress [225]. Additionally, individuals with this condition may display exaggerated distress reactions to unpleasant events [226], have impaired learning abilities [227], exhibit atypical social behaviours [228], experience increased emotional responses and fear-related behaviours [229,230], and show escalating fearful behaviours with age [231].

Furthermore, if these offspring face additional challenges during adulthood, they may become even more vulnerable to adverse health outcomes [232]. Studies have also indicated that they may display behavioural deficiencies and molecular alterations similar to those observed in individuals with schizophrenia [233]. Later, as puppies, social isolation may cause lifelong disturbances and compromised learning abilities [234,235,236].

### 4.3. Lack of Control over Dog Breeding Promotes Criminal Activity

An increased demand for puppies and consumer preferences for designer breeds have led to changes in the dog trade, with the emergence of large producers, international trade, and internet sales [237,238,239] making trafficking an attractive business for mafias [240,241].

A report by the European Commission in the EU has described the illegal activities associated with the dog trade [242]. The position statement notes that a significant proportion of traders undermine EU regulations on the non-commercial transport of pets in order to conceal their true commercial endeavours. Regulation (EU) No 576/2013 allows individuals to travel with up to five animals under certain circumstances without having to register them in TRACES (TRACES, Trade Control and Expert System, is the European Commission’s online platform for sanitary and phytosanitary certification required for the importation of animals, animal products, food and feed of non-animal origin, and plants into the European Union, and the intra-EU trade and EU exports of animals and certain animal products) (Trade Control and Expert System) or show them at BCPs. However, this regulation is being abused by scammers. The second finding of the investigation was that forged and falsified documents were consistently used to accompany the importation of dogs and cats into the European Union from outside. The paperwork appears to be in order at the border crossing. However, it is often impossible to determine their final destination. Then, after being regrouped in the first country of entry, they continue to travel with new documents, posing as EU pets, and their true origins become unknown. Authorities have expressed particular concern for the rabies antibody titration certificate and other issues, including animal health and welfare, breeding conditions, and mutilation of tails, ears, and vocal cords. Illegal trade involves the systematic exploitation of dogs at all levels, from their place of origin and breeding facilities to post-purchase.

Over the last year, there has been a significant increase in online sales [242]. Criminals have exploited this trend, as it is impossible to conduct comprehensive screening, allowing offenders operating remotely to reach a large audience. Online selling and advertising contribute to the loss of traceability, as the information displayed in advertisements is often unverifiable. According to the EU [242], organized networks may sell thousands of animals, with some disguising their actions as rescue efforts. For example, a network of three sellers was reported to offer rescued dogs from all over Europe through more than 1000 unique ads across multiple websites, using just three phone numbers.

Online pet buyers often lack knowledge about the animals they purchase. It can be particularly challenging to determine the true source of a pet due to forged documents and misleading origin claims in advertisements. Although sellers may provide details such as the animal’s microchip number, the parents’ microchip number, and the breeder/seller’s registration number, traceability cannot be ensured without prompt and accurate confirmation of their correspondence. International trade presents a challenge in terms of traceability due to the complex supply chains involving breeders, sellers, and transporters. The cross-border nature of these movements further complicates traceability due to varying legal systems, language barriers, and enforcement capacities across different countries.

Illegal traders are able to transport dogs across borders undetected if pet passports are not securely and irreversibly linked to individual microchips. By attaching the microchip to the animal’s fur instead of injecting it, traceability and health guarantees for the dog are eliminated, making removal simple. This leads to alterations in the ownership, origin, and health information on the passports. Moreover, in many countries, the absence of mandatory dog identification and microchip registration in a database make it easier to obtain animals from unreliable or illegal sources.

Enforcement authorities cannot fully understand the extent of the problem and conduct successful investigations without a comprehensive and reliable database containing accurate and verified information on animals, breeders, and owners. Therefore, it is crucial to acknowledge the global nature of the issue and the illegal online trade of cats and dogs to effectively address the complexities involved.

Furthermore, engaging in criminal activities, such as committing fraud in the pet trade, comes at a surprisingly low cost. This is due to the significant difference between the potential high earnings and the relatively minor penalties imposed. In many cases, fines are relatively low, typically ranging from EUR 100 to EUR 300 for administrative fees per dog. However, even high fines do not deter the trade. The European Commission’s report on illegal trade [242] provides an example of a breeder in Greece who was fined EUR 600,000. However, this did not prevent him from continuing his business.

The European Commission has recently proposed a regulation for the welfare of dogs and cats and their traceability [243]. The proposal includes a new feature that aims to make dogs and cats more traceable, particularly when they are sold or adopted online. According to the proposed regulations, all dogs and cats must be identified by electronically read transponders before they can be sold. This measure is expected to deter fraud and improve the oversight of animal welfare conditions.

## 5. Turning the Screw Even Further: The Unnecessary Cosmetic Modifications

Although practices such as cosmetic surgery, hair colouring, or even tattoos are outside the framework of selective breeding, they are closely related to owners’ and breeders’ preferences for certain aesthetic breed appearances and are therefore also discussed.

### 5.1. Trimming the Dog

Cosmetic surgery may be perceived as less risky than conventional surgery due to the typically young and healthy animals undergoing the procedure. However, these are still surgical interventions and carry the potential for anaesthetic risks, post-operative infections, pain, and the need for additional interventions if the desired outcome is not achieved, with both physiological and psychological impacts on the animal [244,245,246]. Therefore, the prohibition of cosmetic surgery on animals has become increasingly widespread, especially in Europe [247,248]. However, legislative standards vary between countries. In the EU, recent legislation has proposed to extend bans on mutilations through all the member states [243], which are supported and followed by European kennel club standards. However, in other countries, such as the USA, certain ear and tail conformations are required, which can only be achieved by cosmetic surgery. The American Kennel Club states that ‘*Ear cropping, tail docking [and dewclaw], as described in certain breed standards, are acceptable practices integral to defining and preserving breed character*’ [249]. Furthermore, the decision made by owners to modify their animals for aesthetic purposes is not only illegal and ethically questionable, but can also impact their ability to communicate and interact with other animals and humans, compromising their safety and welfare [245,250,251]. It may even affect owners’ abilities to accurately interpret their animals’ emotions, resulting in inappropriate responses to their dogs’ behaviour [252,253].

Tail docking and ear cropping are the most common aesthetic modifications performed on dogs. Throughout history, dogs have had their tails docked for various reasons, including superstitions, supposed health strategies to prevent disease, injury prevention at work, or even as a means of social distinction [254]. The aesthetic justification has been the last to appear, hand in hand with the breed standards [255], becoming standardised in the 20th century.

It is surprising to note that tail docking is not always carried out by a veterinarian, and anaesthesia is not always administered. Studies indicate that only 10% of veterinarians use anaesthetics or painkillers during tail docking [256]. Furthermore, in some countries, breeders and owners are permitted to perform tail docking, which may result in even lower usage of these drugs [257].

Similarly, the justification for ear cropping has evolved from injury prevention to the desired aesthetic appearance according to breed standards, such as the Doberman Pinscher or Pit Bull Terrier.

During the 20th century, interventions to alter the appearance of animals were so widespread that many people were unaware of their supposed benefits or reasons for being carried out. Some even believed that the altered appearance was natural and genetic, rather than the result of surgery [256].

The reasons may not have been clear, but the effects are devastating. Not only does it give dogs a fiercer and more aggressive appearance, but their owners are also perceived to have the same characteristics [256], so it could be considered that owners seek a reflection of these facets in their pets.

Another common surgical procedure is a removal or reduction in the vocal cords to decrease the volume of an animal’s bark, despite the fact that this behaviour can be redirected or corrected through training. In addition to chronic infections, respiratory problems, and pain [258], this procedure significantly impairs the animal’s ability to communicate. Devocalization does not eliminate the motivation or the behaviour itself; it only reduces the volume of barking. Instead of searching for the cause of the barking and addressing it, the animal is mistreated to prevent irritating the owner [259]. The reality is that dogs bark. It is a species-specific trait that has been valued by humans for thousands of years for its role in alarming animals or strangers [260], but dogs can be trained to communicate silently without being devocalized.

In 1987, the EU launch the European Convention for the Protection of Pet Animals (ETS No. 125) [247], banning ear cropping, declawing, and tail docking, with a “*Resolution on surgical operations in pet animals*” in 1995 “*to promote awareness particularly among judges, breeders, veterinarians and keepers that [aesthetics] mutilation should not be carried out [and] to encourage breeding associations to amend breeding standards*” [261] (p. 2). An EU-wide ban on ear cropping and tail docking, which are still allowed in a small number of member states, is also part of the Commission’s recent legislation proposal [243].

Some other cosmetic surgeries are disguised as corrective surgeries, as they claim to address issues that arise as a result of diseases contracted by the animal. For instance, certain eye pathologies necessitate the removal of the eyeball, which can leave the animal with an appearance that may be shocking or unpleasant for its caregivers. In such cases, a silicone prosthesis may be inserted into the eye socket to enhance the animal’s appearance [262]. Similarly, testicular prostheses have been introduced in males to maintain the original aesthetic appearance after castration [263]. So, these interventions should be considered purely aesthetic because they do not provide any functional, physical, or psychological benefit to the animal. They may only be justified in preventing the breakdown of the human–animal bond, particularly when children are involved.

Other surgeries such as correcting skin wrinkles to prevent recurring skin infections, or correcting eyelids to avoid eye injuries, or performing soft palate surgeries in brachycephalic dogs with severe respiratory problems, which are in fact corrective surgeries, raise concerns as well. Nevertheless, they do not stem from the surgeries themselves, but rather from the fact that these issues could have been addressed at the breed level by modifying official standards.

### 5.2. Mirroring Humans: Hair Dye, Tattoos, and the Like

Humans use their appearance as a form of expression and communication to the world around them. However, extending this communicative function to animals can be problematic. Some owners choose to dye their animals’ hair, just as they would change their own hairstyle or hair colour. This is often performed for fun or economic reasons, but owners may not be aware that this practice can cause multiple respiratory problems [264] and hepatic damage [265,266].

Other forms of human expression include tattoos or piercings on different parts of the body, which can also be extended to our pets. These interventions should be considered acts of animal abuse, as they are painful procedures not required by the animals. They are usually performed without anaesthesia, increase the likelihood of infections [267], and can cause hypersensitivities [268] and self-mutilation through excessive licking.

## 6. Conclusions

The changing preferences and participation of dogs in today’s society, the lack of ethics in breeding and trade that prioritise animal health and welfare over economic benefit or mere aesthetic appearance, or the lack of real control over new marketing channels are putting animal health and welfare at risk.

A breeding system that solely focuses on selling price, supply and demand, or corporate profits systematically ignores health problems that could be genetically controlled or avoided, as well as behavioural problems that may arise from breeding and selling conditions or be conditioned by the genetic selection of parents. Kennel clubs, responsible for recognizing and registering purebred individuals, should not ignore this reality and must take a stance, as they are already doing so in the European Union, Norway, and the United Kingdom. Legislators should increase their awareness of the exploitation and abuse of animals in breeding and the pet trade, particularly in relation to puppy farms, internet sales, and international trade. They should promote regulations that effectively protect animals and reassure future owners that they are not contributing to animal suffering.Prospective pet owners should consider the options of adoption versus purchase to avoid contributing to an already exacerbated demand, and ultimately to responsibly ensure the conditions under which the animal they are buying has been bred and kept.

Although some countries such as Norway, the United Kingdom, and the European Union have recently introduced legislative initiatives to control breeding, trade, and aesthetic mutilations, these concerns are not yet widely shared among legislators, breeders, breed associations, let alone the general public. To curb the interest that criminal gangs are finding in this lucrative business, public registries of animals, breeders, and traders should be shared or made accessible by authorities in different countries. Additionally, the extension of unique microchip identification of animals and standardisation of legislation for transport and trade between countries would be beneficial.

However, perhaps the key is to influence the actors that determine market demand, acting on the information that veterinarians provide about the different breeds, breeders, and origins of the animals to be purchased, as well as on the media that can, unintentionally we assume, drive people crazy about a certain breed. This would extend the responsibility of the new owner beyond the current and future welfare of their newly acquired animal by making them aware of the origins, processes, and consequences of certain breeding and trading systems on the health and welfare of thousands of animals.

## Figures and Tables

**Table 1:** Examples of inherited disorders in popular dog breeds.

Breed	Inherited Disorder	Reference
German Shepherd	Hip dysplasia Exocrine pancreatic insufficiency Degenerative lumbosacral stenosis	[48,49] [50,51] [52,53]
Labrador Retriever	Canine atopic dermatitis Tricuspid valve malformation Copper associated chronic hepatitis	[54] [55,56] [57,58]
Jack Russell Terrier	Legg-Calve-Perthes disease Epilepsy Pulmonic stenosis	[59] [60] [61]
French Bulldog	Brachycephalic obstructive airway syndrome (BOAS) Dystocia Corneal ulceration	[62,63] [64] [65]
Golden Retriever	Hip dysplasia Elbow dysplasia Heart conditions	[66,67] [68,69] [70,71]
Bulldog	Skin fold dermatitis Prolapsed nictitating membrane gland Protruding lower jaw Brachycephalic obstructive airway syndrome (BOAS)	[72] [73] [74] [63]
Cocker Spaniel	Nephropathy Glaucoma Distichiasis	[75,76] [77] [78,79]
Beagle	Skin problems Neonatal cerebellar cortical degeneration Canine degenerative myelopathy	[80] [81] [82]
Rottweiler	Unilateral cranial cruciate ligament rupture Canine degenerative myelopathy Elbow dysplasia	[83] [84] [85]
Dachshund	Intervertebral disk disease Heart conditions Chronic enteropathy	[39,86,87] [88] [89]
Cavalier King Charles spaniel	Fly catching Idiopathic epilepsy Idiopathic facial nerve paralysis with or without associated vestibular disease Degenerative myelopathy	[90] [91] [92] [82]
Pugs and Basenjis	Hemivertebrae and spina bifida	[47,93]
Rhodesian Ridgeback	Dermoid sinus Arrhythmia and sudden death	[94] [95]
Dalmatians	Deafness	[96,97]
